# Contributions of elevated CRP, hyperglycaemia, and type 2 diabetes to cardiovascular risk in the general population: observational and Mendelian randomization studies

**DOI:** 10.1186/s12933-024-02207-0

**Published:** 2024-05-10

**Authors:** Monica G Rolver, Frida Emanuelsson, Børge G Nordestgaard, Marianne Benn

**Affiliations:** 1 Department of Clinical Biochemistry, Copenhagen University Hospital - Herlev Gentofte, Borgmester Ib Juuls Vej 1, Herlev, 2730 Denmark; 2 Institute of Clinical Medicine, Faculty of Health and Medical Sciences, University of Copenhagen, Blegdamsvej 3B, Copenhagen, 2200 Denmark; 3 Department of Clinical Biochemistry, Copenhagen University Hospital - Herlev Gentofte, Borgmester Ib Juuls Vej 1, Herlev, 2730 Denmark; 4 The Copenhagen General Population Study, Copenhagen University Hospital - Herlev Gentofte, Borgmester Ib Juuls Vej 1, Herlev, 2730 Denmark

**Keywords:** Hyperglycaemia, Diabetes, Low-grade inflammation, Cardiovascular disease, Cardiovascular death, Mendelian randomization

## Abstract

**Objective:**

To investigate the contributions of low-grade inflammation measured by C-reactive protein (CRP), hyperglycaemia, and type 2 diabetes to risk of ischemic heart disease (IHD) and cardiovascular disease (CVD) death in the general population, and whether hyperglycaemia and high CRP are causally related.

**Research design and methods:**

Observational and bidirectional, one-sample Mendelian randomization (MR) analyses in 112,815 individuals from the Copenhagen General Population Study and the Copenhagen City Heart Study, and bidirectional, two-sample MR with summary level data from two publicly available consortia, CHARGE and MAGIC.

**Results:**

Observationally, higher plasma CRP was associated with stepwise higher risk of IHD and CVD death, with hazard ratios and 95% confidence intervals (95%CI) of 1.50 (1.38, 1.62) and 2.44 (1.93, 3.10) in individuals with the 20% highest CRP concentrations. The corresponding hazard ratios for elevated plasma glucose were 1.10 (1.02, 1.18) and 1.22 (1.01, 1.49), respectively. Cumulative incidences of IHD and CVD death were 365% and 592% higher, respectively, in individuals with both type 2 diabetes and plasma CRP ≥ 2 mg/L compared to individuals without either. Plasma CRP and glucose were observationally associated (β-coefficient: 0.02 (0.02, 0.03), *p* = 3 × 10^− 20^); however, one- and two-sample MR did not support a causal effect of CRP on glucose (−0.04 (−0.12, 0.32) and − 0.03 (−0.13, 0.06)), nor of glucose on CRP (−0.01 (−0.08, 0.07) and − 0.00 (−0.14, 0.13)).

**Conclusions:**

Elevated concentrations of plasma CRP and glucose are predictors of IHD and CVD death in the general population. We found no genetic association between CRP and glucose, or vice versa, suggesting that lowering glucose pharmacologically does not have a direct effect on low-grade inflammation.

**Supplementary Information:**

The online version contains supplementary material available at 10.1186/s12933-024-02207-0.

## Highlights

Hyperglycaemia and low-grade inflammation are both associated with type 2 diabetes and cardiovascular disease (CVD). Causality between plasma C-reactive protein (CRP) and glucose concentrations has not been determined. Clarifying causality might direct opportunities in prevention and treatment of diabetes and CVD.

We investigated whether plasma concentrations of CRP and glucose were causally associated and their associations with risk of ischemic heart disease (IHD) and CVD death.

Elevated plasma CRP and hyperglycaemia were both associated with increased risk of IHD and CVD death.

Plasma CRP and glucose concentrations were not causally associated.

**Supplementary Information:**

The online version contains supplementary material available at 10.1186/s12933-024-02207-0.

## Introduction

Type 2 diabetes, characterized by hyperglycaemia, affects millions of people worldwide and is a major risk factor of cardiovascular disease [1–3]. Chronic low-grade inflammation is a common feature in individuals with type 2 diabetes and is associated with high risk of atherosclerotic cardiovascular disease (CVD) [4–10]. Hyperglycaemia has been shown to associate with increased concentrations of inflammatory markers such as plasma C-reactive protein (CRP) [11–14]. In turn, CRP and other proinflammatory markers have been associated with increased risk of hyperglycaemia and type 2 diabetes, as well as with ischemic heart disease (IHD) and CVD death [4, 15, 16]. Thus, low-grade inflammation has been proposed as a potential mechanism linking type 2 diabetes and cardiovascular disease. However, the extent to which low-grade inflammation and type 2 diabetes, independently and combined, add to the risk of IHD and CVD death is not known. Furthermore, it is not clear whether hyperglycaemia causally contributes to low-grade inflammation, whether low-grade inflammation casually contributes to hyperglycaemia, or both [17–20]. This is important, as ongoing randomized controlled trials of glucose-lowering drugs, i.e. semaglutide, are suggested to lower inflammation [17]. Also, several prospective studies have shown that individuals with higher CRP levels have higher risk of incident type 2 diabetes [21–23], indicating that low-grade inflammation may be a cause of diabetes. Conversely, so far, drugs targeted to lower inflammation has shown no effect on type 2 diabetes incidence [24].

Mendelian randomization (MR) is an epidemiological approach combining genetic information with traditional epidemiological methods to provide less confounded estimates compared to observational associations [25]. MR uses genetic variants strongly associated with a specific phenotype to compare the risk of an outcome in population subgroups stratified by the genotype. Due to the independent segregation of alleles from parents to offspring at conception, the population subgroups by genotype will differ systematically only in terms of the specific phenotype and confounding factors will be largely evenly distributed [25].

We investigated the contributions of type 2 diabetes and CRP ≥ 2 mg/L to the risk of IHD and CVD death and the potential causal relationship between hyperglycaemia and elevated CRP, using (i) observational analyses and bidirectional, one-sample MR analyses in 112,815 individuals from the Copenhagen City Heart Study and the Copenhagen General Population Study, and (ii) bidirectional two-sample MR analyses in up to 165,518 individuals using publicly available data on fasting glucose concentrations from the Meta-Analyses of Glucose and Insulin-related traits Consortium (MAGIC) [26] and on CRP concentrations in 204,402 individuals of European ancestry from the Cohorts for Heart and Aging Research in Genomic Epidemiology (CHARGE) Inflammation Working Group (CIWG) [27] (Supplementary Fig. 1).

## Materials and methods

### Study populations

#### Copenhagen studies

We included 112,815 individuals from the Copenhagen studies: the Copenhagen City Heart Study (CCHS) and the Copenhagen General Population Study (CGPS) for observational, one-sample, and two-sample (as a sensitivity analysis), bidirectional Mendelian randomization analyses. The CCHS and CPGS are both prospective population-based studies approved by the Danish National Committee on Health Research Ethics (approval numbers: H-KF-01-144/01, KF-100.2039/91, KF-01-144/01). Both were conducted according to the Declaration of Helsinki and written informed consent was obtained from all individuals. All individuals were white and of Danish descent and none were included in more than one study [28]. The CCHS was initiated in 1976 to 1978 with follow-up examinations in 1981 to 1983, 1991 to 1994, and 2001 to 2003. Individuals were selected at random from the national Danish Civil Registration System to reflect the Danish general population aged 20 to 100 + years. Linked to Danish health registries the follow-up time is up to 40 years. The CGPS was initiated in 2003 with enrolment until 2015 and ongoing follow-up examinations [4]. Participants in both studies filled in a questionnaire, which was reviewed with an examinator on the day of attendance. They also completed a physical examination and had a blood sample drawn [28]. 3,608 participants were excluded due to missing data on entry measurements of plasma glucose and CRP. Furthermore, 594 participants were excluded as they were registered with type 1 diabetes, resulting in a total of 4,202 individuals being excluded, corresponding to 3.5% of the cohort.

#### MAGIC and CHARGE CIWG consortia

Publicly available summary data for the selected genotypes were included for two-sample, bidirectional Mendelian randomization analyses. Beta-coefficients for fasting glucose concentrations from 165,518 to 200,392 individuals without diabetes and of European ancestry were used from the Meta-Analyses of Glucose and Insulin-related traits Consortium (MAGIC) [29]. Beta-coefficients for CRP concentrations in 204,402 individuals of European ancestry were used from the Cohorts for Heart and Aging Research in Genomic Epidemiology (CHARGE) Inflammation Working Group (CIWG) [27]. The MAGIC and CHARGE CIWG had partially overlapping sets of participants, accounting for up to 80% of the MAGIC participants and up to 65% of the CHARGE CIWG participants. Detailed information regarding overlapping participants is shown in Supplementary Table 1.

### Genotypes

A targeted selection approach of genetic variants was chosen to reduce the risk of pleiotropy [30, 31]. Six genetic variants with well-established associations with high glucose and HbA_1c_ concentrations in genome wide association studies were selected as genetic instruments (rs560887, rs2191349, rs4607517, rs7903146, rs10811661, and rs11708067) [28, 32, 33]. The variants were selected as they represent independent loci showing strong associations between the genotype and glycemia and in genes known to be involved in glucose metabolism (Supplementary Table 2). The variants were not in linkage disequilibrium (R^2^ = 0.004). Three genetic variants known to be associated with CRP in genome wide association studies were selected as genetic instruments (rs1205, rs3093077, and rs1130864) [4, 15, 34]. Theses variants are located in the *CRP* gene and were not in linkage disequilibrium (R^2^ < 0.2) Detailed information regarding the genetic instruments are shown in Supplementary Table 3. Information regarding linkage disequilibrium was retrieved from the web-based application LD-link [35]. In the Copenhagen studies, the variants were genotyped using an ABI PRISM 7900HT Sequence Detection System (Applied Biosystems Inc., Foster City, CA) and TaqMan-based assays. Large-scale sequencing (genotype arrays) in all individuals have not been performed in the Copenhagen studies.

Genetic variants for CRP and glucose were each combined into a weighted allele score, weighted by effect size on phenotype, i.e. plasma CRP and glucose, and allele frequency in the CCHS and CGPS combined. Only individuals with genotypes available for all variants of either CRP or glucose were included in genetic analyses, resulting in 105,759 individuals with a weighted allele score for CRP and 106,458 individuals with a weighted allele score for glucose (Supplementary Table 4).

### C-reactive protein, glucose, and covariates

Blood samples were taken non-fasting and were measured fresh using standard hospital assays. Plasma CRP concentrations were measured using high sensitivity turbidimetry while plasma glucose, triglycerides, and high- and low-density lipoprotein cholesterol were measured by colorimetric assays. Blood samples were taken at random irrespective of time since and content of the last meal. Body mass index (BMI) was calculated by measured weight in kilograms divided by measured height in meters squared. Blood pressure was measured with automated equipment. Information on alcohol consumption in units per week, smoking, physical activity, and use of lipid-lowering medication were self-reported. Smoked pack-years were calculated as accumulated time of tobacco smoking and consumed tobacco per day in current and former smokers. Physical activity was categorized as low, medium, and high activity during work and leisure time.

### Type 2 diabetes, ischemic heart disease, and cardiovascular death

Diagnoses of type 2 diabetes, ischemic heart disease (IHD) and cardiovascular disease death (CVD death) were as defined by the World Health Organization (WHO) International Classification of Disease (ICD)-10 and 8 codes (Supplementary Table 5). All diagnoses in the national Danish Patient Registry and the national Danish Cause of Death Registry are recorded by physicians in compliance with national laws. We did not lose track of any individual, as all individuals living in Denmark have a Danish Civil Registration System number, which is 100% complete including death and emigration.

### Statistical analyses

We used Stata SE 17.0 for Windows (StataCorp). Deviation from Hardy-Weinberg equilibrium was investigated using chi-square tests. Test for trend across ordered weighted allele score categories for plasma CRP and glucose were by the non-parametric Cuzick’s extension of a Wilcoxon rank sum test. The population distribution of plasma CRP by diabetes-status and the population distribution of plasma glucose by CRP concentrations  <  or ≥ 2  mg/L were plotted using the *kdensity* function in Stata. To compare medians of plasma CRP and glucose between groups we used the non-parametric equality-of-medians test. CRP concentrations were not normally distributed and therefore log-transformed before incorporated into the regression models. The observational association between CRP and glucose was investigated using linear regression adjusted for age, sex, BMI, alcohol consumption, smoking status, physical activity, blood pressure, low-density lipoprotein cholesterol, triglycerides, and lipid-lowering medication and was graphically displayed using kernel-weighted local polynomial smoothing and geometric means with 95% confidence intervals. The same analyses were also performed stratified by sex and age.

Multivariable Cox proportional hazards regression models were used to estimate hazard ratios for the associations of plasma CRP and glucose concentrations with risk of IHD and CVD death. Graphs were truncated at glucose concentrations of 2.5 mmol/L (45 mg/dL) and individuals with lower concentrations were excluded from the Cox regression analyses because of a low number of individuals. The group with plasma glucose between 4.7 mmol/L (85 mg/dL) and 5.0 mmol/L (90 mg/dL) was used as reference group due to the normal physiological concentrations [36]. The models were adjusted for age, sex, BMI, alcohol consumption, smoking status, physical activity, blood pressure, low-density lipoprotein cholesterol, triglycerides, and lipid-lowering therapy.

The cumulative incidence of type 2 diabetes, IHD and CVD death as a function of age was plotted using a Kaplan-Meier estimator approach comparing the population divided into groups. The groups were categorized by diabetes-status (*type 2 diabetes* or *no type 2 diabetes*) and plasma CRP concentrations (*CRP < 2 mg/*L or *CRP ≥ 2mg/L*). Log-rank tests were used to test for differences between groups. Cumulative incidence of CVD death was also estimated using competing risk regression based on Fine and Gray proportional sub-hazards model [37] accounting for the possibility of death from other cause as a competing event (Supplementary Fig. 2).

We next tested whether the selected genetic variants and the weighted allele score were associated with plasma CRP and glucose concentrations in the Copenhagen studies and used instrumental variable analysis with two-stage least-squares regression in bidirectional one-sample Mendelian randomization analysis to study the causal association between plasma CRP and plasma glucose, and vice versa, using the ivreg2 command. The strength of the genetic instrument (i.e. the association between weighted allele scores and glucose concentrations) was confirmed by F statistics in the first-stage regression [25, 38]. Estimates were reported as average difference per 1 mg/dL higher CPR and 1 mmol/L (18 mg/dL) higher glucose concentration, respectively. The genetic analyses were not adjusted for any covariates as the allele scores are largely unconfounded. For independent confirmation, we also performed bidirectional two-sample Mendelian randomization analyses with publicly available data from the MAGIC and CHARGE consortia, using the same genetic variants for plasma CRP and glucose as in the one-sample analyses in the Copenhagen studies. The beta-coefficients for instrumented CRP and instrumented glucose, respectively, were combined using instrumental variable analyses with inverse variance weighted (IVW) regression. Sensitivity analyses accounting for direct pleiotropic effects (MR Egger) and for up to 50% of information coming from invalid or weak instruments (weighted median) were also performed using the publicly available, user-written *mrmedian* and *mregger* commands [39, 40]. Heterogeneity was tested for using Cochran’s Q test and reported as I^2^, where an I^2^ between 0 and 40% is considered low, between 40 and 60% as moderate and above 60% as high [41].

## Results

Baseline characteristics for all individuals, and for individuals with and without type 2 diabetes are shown in Supplementary Table 6. Individuals with type 2 diabetes were older, more often women, had a higher BMI, and were more likely to be physically inactive compared to individuals without type 2 diabetes.

### Observational associations between plasma glucose and C-reactive protein

**Fig. 1:**
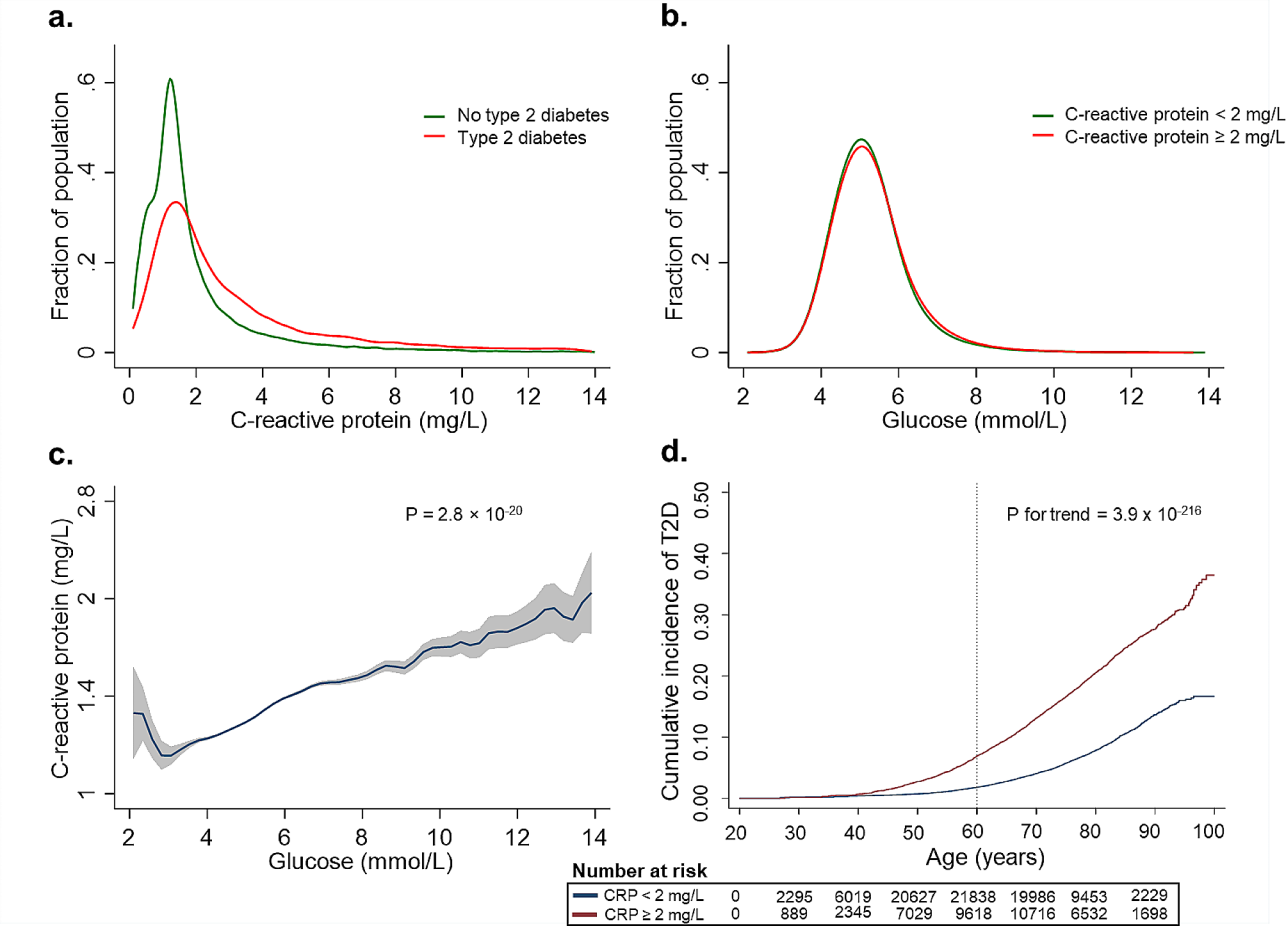
Interrelationships between plasma C-reactive protein, plasma glucose, and diabetes in 110,746 individuals in the Copenhagen studies. **(a)** Population distribution of plasma C-reactive protein (CRP) in individuals with (red line) and without (green line) type 2 diabetes. **(b)** Population distribution of plasma glucose in individuals with a plasma CRP < 2 mg/L (green line) or a CRP ≥ 2 mg/L (red line). **(c)** Correlation between plasma CRP (blue line) and glucose concentration, adjusted for age, sex, body mass index, alcohol consumption, smoking status, physical activity, blood pressure, low-density cholesterol, triglycerides, and lipid-lowering therapy and displayed using kernel-weighted local polynomial smoothing and geometric means with 95% confidence intervals (grey area). **(d)** Cumulative incidence of type 2 diabetes as a function of age. The population was divided into two groups: CRP < 2 mg/L (blue line) and CRP ≥ 2 mg/L (red line). 1 mmol/L of glucose is equivalent to 18 mg/dL

Figure 1 shows the population distribution of plasma CRP in individuals with and without type 2 diabetes (a.), the population distribution of plasma glucose in individuals with plasma CRP < or ≥ 2 mg/L (b.), the multivariable adjusted correlation of plasma glucose as a function of plasma CRP (c.) and the cumulative incidence of type 2 diabetes stratified by plasma CRP < or ≥ 2 mg/L (d.). Median plasma CRP was 2.1 mg/L in individuals with type 2 diabetes and 1.4 mg/L in individuals without diabetes (*p* = 7 × 10^− 264^) (a.). Median plasma glucose was 5.1 mmol/L (92 mg/dL) in individuals with a plasma CRP < 2 mg/L and 5.3 mmol/L (95 mg/dL) in individuals with a plasma CRP ≥ 2 mg/L (*p* = 6 × 10^− 146^) (b.). In the multivariable adjusted model, there was a linear relationship between higher plasma CRP and higher plasma glucose (*p* = 3 × 10^− 20^) (c.). The cumulative incidence of type 2 diabetes stratified by plasma CRP showed a gradually increasing risk of type 2 diabetes from age 40 in individuals with CRP ≥ 2 mg/L compared to CRP < 2 mg/L (d.).

For both plasma CRP and glucose, there was a linear association with higher age. Stratifying the analyses for sex showed higher plasma CRP with increasing plasma glucose concentrations in men compared to women (Supplementary Fig. 3).

### Risk of ischemic heart disease and cardiovascular death

**Fig. 2:**
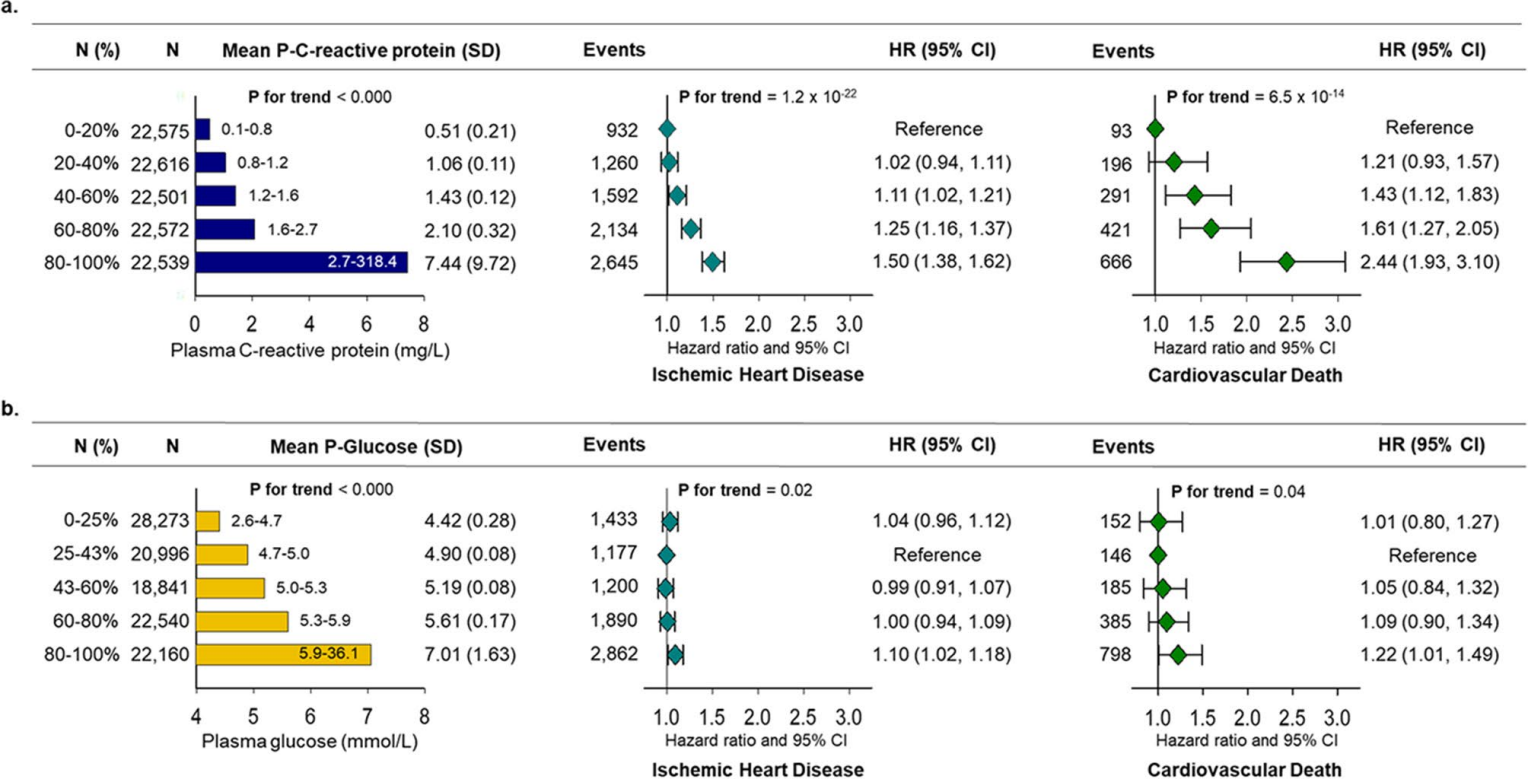
Mean plasma concentrations of C-reactive protein and glucose and hazard ratios (HRs) for ischemic heart disease (IHD) and cardiovascular disease (CVD) death in 112,815 individuals in the Copenhagen studies. Plasma C-reactive protein concentrations (**a.**) and plasma glucose concentrations (**b.**) are shown in quintiles. Individuals with a plasma glucose concentration below 2.5 mmol/L (45 mg/dL) were excluded. Risk estimates by Cox regression analyses were adjusted for age, sex, body mass index, alcohol consumption, smoking status, physical activity, blood pressure, low-density cholesterol, triglycerides, and lipid-lowering therapy. 1 mmol/L of glucose is equivalent to 18 mg/dL. SD = standard deviation, CI = confidence interval

Figure 2 shows the prospective risk of IHD and CVD death as a function of higher CRP (a.) and glucose (b.) concentrations in quintiles. There was a stepwise increase in risk of IHD with hazard ratios (HR) in the 3rd-5th CRP quintiles from 1.11 (95% confidence interval: 1.02, 1.21) to 1.50 (1.38, 1.62) compared to the lowest quintile. Similarly, there was a stepwise increase in risk of CVD death with HR in the 3rd-5th CRP quintiles from 1.43 (1.12, 1.83) to 2.44 (1.93, 3.10). Overall risk of IHD and CVD death increased with increasing plasma CRP concentrations (p for trend = 1 × 10^− 22^ and 7 × 10^− 14^).

In contrast, only a minor stepwise increased risk of IHD and CVD death was seen for the 3rd-5th glucose quintiles (p for trend = 0.02 and 0.04) with only individuals in the 5th glucose quintile being at increased risk of IHD and CVD death with HRs of 1.10 (1.02,1.18) and 1.22 (1.01,1.49).

**Fig. 3:**
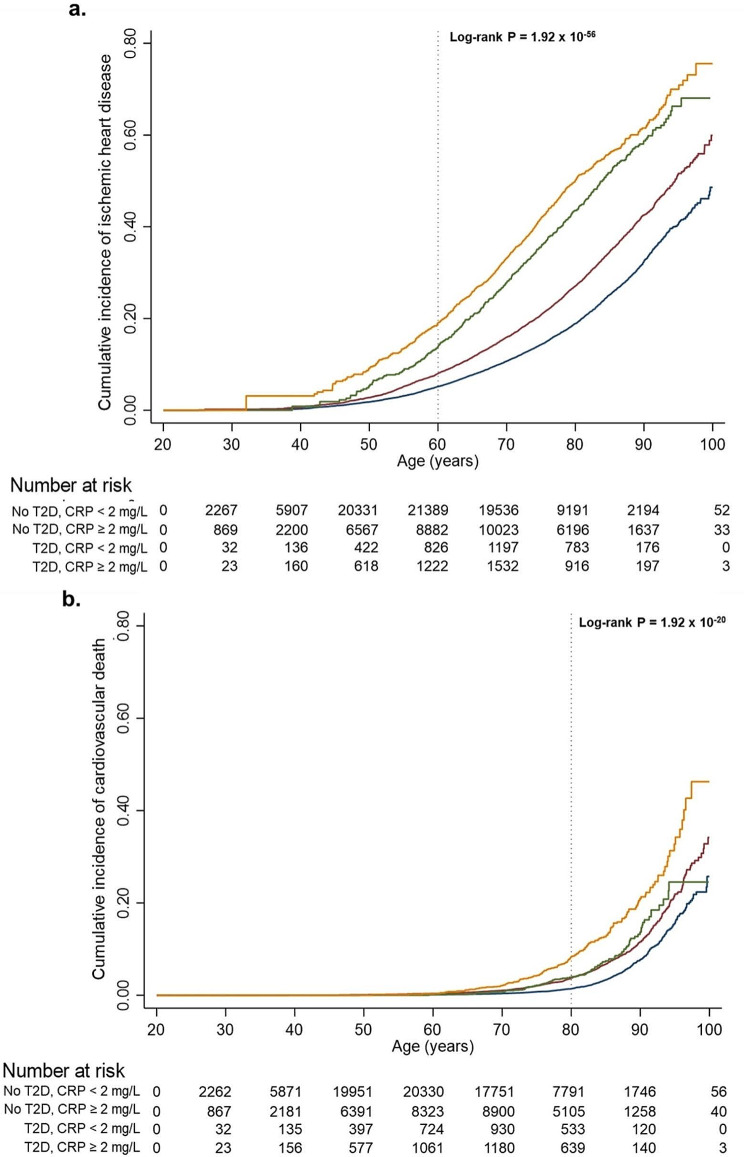
Cumulative incidence of ischemic heart disease and cardiovascular disease death in 112,815 individuals in the Copenhagen studies. Cumulative incidence of ischemic heart disease (IHD; **a.**) and cardiovascular disease death (CVD death; **b.**) as a function of age and plotted as a Kaplan-Meier estimator. The population was divided into four categories: no type 2 diabetes (T2D) and C-reactive protein (CRP) < 2 mg/L (blue line); no T2D and CRP ≥ 2 mg/L (red line); T2D and CRP < 2 mg/L (green line); and T2D and CRP ≥ 2 mg/L (yellow line). Log-rank tests were used to examine for differences among ordered categories

Figure 3 shows the cumulative incidence of IHD (a.) and CVD death (b.) as a function of age and according to type 2 diabetes and low-grade inflammation status (CRP < or ≥ 2 mg/L). The cumulative incidence of IHD and CVD death increased stepwise from individuals with no type 2 diabetes and CRP < 2 mg/L, through individuals with no type 2 diabetes and a CRP ≥ 2 mg/L, individuals with type 2 diabetes and a CRP < 2 mg/L, and to individuals with both type 2 diabetes and a CRP ≥ 2 mg/L (p for trend = 2 × 10^− 56^ and 2 × 10^− 20^). At age 60 years, the lowest and highest risk (no type 2 diabetes and a CRP < 2 mg/L and both type 2 diabetes and a CRP ≥ 2 mg/L) categories had cumulative incidences of 5.2% and 19.0% for IHD, respectively, equivalent to a 365% increase in risk of IHD. At age 80, the corresponding lowest and highest risk factor scoring groups had cumulative incidences of 1.4% and 8.3% for CVD death, respectively, equivalent to a 592% increase in risk of CVD death.

### Genetic associations between plasma glucose and C-reactive protein

**Fig. 4:**
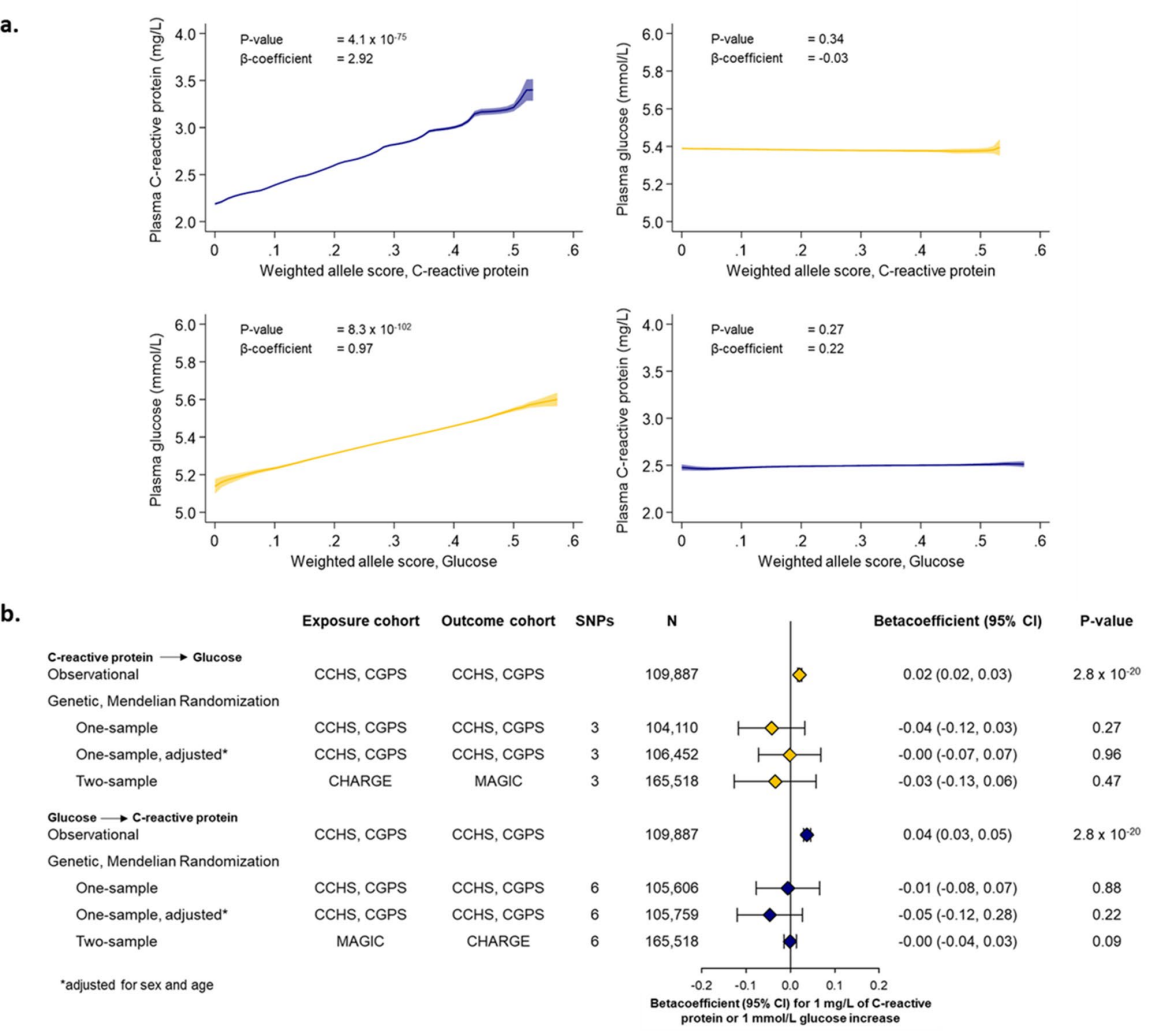
Genetic Mendelian randomization analyses in the Copenhagen studies and in CHARGE and MAGIC consortia. **(a)** Plasma C-reactive protein (CRP) and glucose concentrations as a function of, respectively, the weighted CRP and the glucose allele scores as continuous variables. The weighted CRP allele score was generated using the genetic variants rs1205, rs1130864, and rs3093077, located in the *CRP* gene. The weighted glucose allele score was generated using the genetic variants rs560887, rs2191349, rs4607517, rs7903146, rs10811661, and rs11708067. **(b)** Observational and Mendelian randomization analyses of the effect of a 1 mg/L higher plasma CRP on plasma glucose, or the effect of a 1 mmol/L (18 mg/dL) higher glucose on CRP. Observational results were obtained using multifactorially adjusted linear regressions and with 95% confidence intervals. Instrumental variable analyses with two-stage least-squares regression were used in one-sample Mendelian randomization. Inverse-variance weighted analyses were performed with publicly available data from the CHARGE and MAGIC consortia in two-sample Mendelian randomization. 1 mmol/L of glucose is equivalent to 18 mg/dL. CI = confidence interval, CCHS = Copenhagen City Heart Study, CGPS = Copenhagen General Population Study, CHARGE = The Cohorts for Heart and Aging Research in Genomic Epidemiology, MAGIC = the Meta-Analyses of Glucose and Insulin-related traits Consortium

Figure 4a shows plasma CRP and glucose concentrations as a function of the genetic CRP and glucose allele scores on a continuous scale. The selected genetic variants for CRP and glucose were as weighted allele scores associated with respectively continuously higher plasma CRP and continuously higher glucose concentrations. The beta-coefficient of the regression of plasma CRP on CRP alleles was 2.92 (*p* = 4.1 × 10^− 75^) and the regression of plasma glucose on glucose alleles was 0.97 (*p* = 8.3 × 10^− 102^). The genetic CRP score explained 0.3% of the variation in plasma CRP in the population with an F-value = 445 (F > 10 indicates sufficient strength to ensure statistical reliability of the instrumental variable estimates [38, 40]). The genetic glucose score explained 0.4% of the variation in plasma glucose in the population with an F-value = 1,255.

Importantly, the CRP allele score did not associate with plasma glucose concentration (p = 0.34) and the glucose allele score did not associate with plasma CRP concentration (p = 0.27). The associations were also estimated with the mean plasma CRP and glucose concentrations as a function of the genetic CRP and glucose allele scores divided into quintiles showing similar results (Supplementary Fig. 4).

### Observational versus genetic analyses

Figure 4b shows the observational analyses, where a 1 mg/L higher plasma CRP level was associated with a 0.02 mmol/L (0.02, 0.03) (0.36 mg/dL) higher plasma glucose(*p* = 3 × 10^− 20^). A 1 mmol/L (18 mg/dL) higher plasma glucose level was associated with 0.04 mg/L (0.03, 0.05) higher plasma CRP(*p* = 3 × 10^− 20^). In the one-sample Mendelian randomization analyses, a 1 mg/L genetically determined higher plasma CRP level was not associated with plasma glucose (*p* = 0.27) and a 1 mmol/L (18 mg/dL) genetically determined higher plasma glucose level was not associated with plasma CRP (*p* = 0.88). Similarly, in the two-sample Mendelian randomization analyses, a 1 mg/L genetically determined higher plasma CRP concentration was not associated with plasma glucose (*p* = 0.47). A 1 mmol/L (18 mg/dL) genetically determined higher plasma glucose level was not associated with plasma CRP (*p* = 0.09). MR Egger and weighted median regressions showed similar results, with no indication of pleiotropy or heterogeneity (Supplementary Table 7) and two-sample MR within the Copenhagen studies showed similar results, also with no indication of pleiotropy or heterogeneity (Supplementary Table 8).

## Discussion

In this general population cohort, we found an observational association between elevated plasma C-reactive protein and glucose. We also found that CRP ≥ 2 mg/L associates with increased risk of type 2 diabetes, and that CRP ≥ 2 mg/L and type 2 diabetes, individually and jointly, contribute to stepwise higher risk of ischemic heart disease and cardiovascular death. However, bidirectional one- and two-sample genetic Mendelian randomization analyses did not support causality between CRP and glucose, indicating that inflammation and type 2 diabetes are correlated but independent risk factors of cardiovascular disease and death. These genetic findings are novel.

Several other studies have investigated the relationship between inflammatory markers such as CRP and IL-6, and glycaemic traits and type 2 diabetes using MR, however, the results are inconsistent [17, 20, 42–48]. A two-sample MR study investigated the genetic associations between type 2 diabetes, glycaemic traits, and CRP using 15 genetic variants associated with CRP, with 6 variants located in the *CRP* gene, and found a potential causal effect of CRP on risk of type 2 diabetes [42]. A smaller one-sample MR study found no causal effect of CRP on hyperglycaemia or insulin resistance, as measured by HbA1c and HOMA-IR [47]. The authors pointed to upstream inflammatory effectors as possible causal mediators as they observed a clear association between CRP and incident type 2 diabetes, but no indication of causality.

The effects of anti-inflammatory drugs on plasma glucose concentrations and the effects of glucose-lowering drugs on inflammatory markers have also been investigated. A recent exploratory analysis of four different phase 3 trials from the clinical development programs of both subcutaneous and oral semaglutide [17], a glucagon like peptide-1 receptor agonist, showed that semaglutide reduced CRP compared to comparator drugs in individuals with type 2 diabetes. In mediation analyses, the reduction was partially attributed to improved glycaemic control, which is not supported by our findings, and weight loss. The authors also suggested a direct effect of semaglutide on CRP. A randomized controlled trial(RCT) tested the effect of long-term anti-inflammatory treatment with low-dose aspirin and found no effect on risk of type 2 diabetes in women [49]. Another RCT tested the potential effects of canakinumab, an anti-inflammatory drug targeting interleukin-1β proven to prevent cardiovascular events, on fasting plasma glucose, HbA_1c_, and risk of type 2 diabetes in individuals with prediabetes, previous myocardial infarction, and CRP > 2 mg/L [50]. The trial found an increased risk of type 2 diabetes with higher concentrations of CRP and IL-6. Canakinumab treatment reduced CRP and IL-6 concentrations, however, there was no effect on HbA_1c_ or risk of new-onset type 2 diabetes, supporting that low-grade inflammation is not causal for type 2 diabetes and that this treatment will not have effect on type 2 diabetes. Post hoc analyses from the same trial found that treatment with canakinumab reduced cardiovascular risk to a similar extent in patients with and without type 2 diabetes [24]. The implications of these findings are that reducing inflammation with drugs targeting inflammation, such as canakinumab may reduce risk of cardiovascular disease but not type 2 diabetes mellitus.

An observational study recently found an increased risk of vascular mortality in patients with type 2 diabetes and with CRP concentrations of 3.2–10 mg/L [16]. In the present study, we found increased risk of CVD death from mean plasma CRP concentrations above 1.4 mg/L, regardless of diabetes status (Fig. 2). The cumulative incidence of CVD death at age 80 years was comparable in individuals with a CRP ≥ 2 mg/L or with type 2 diabetes (3.7% or 4.0%, respectively). If both risk factors were present the cumulative incidence of CVD death was more than doubled(8.3%).

### Strengths and limitations

The strengths of the present study include the comprehensive observational and bidirectional, one- and two-sample MR design. Investigating the risk of IHD and CVD death with a long follow-up period and potential bidirectional causality in the same cohort as well as with a two-sample design using publicly available summary data from two consortia, strengthens the results and reduces risk of confounding and pleiotropy. Furthermore, due to the updated Danish registries, no individuals were lost during follow-up and all deaths are confirmed.

Potential limitations to the MR analyses include population stratification, weak instrument bias and potential pleiotropic effects [51, 52]. Our cohorts were ethnically homogenous, all individuals being white and of Danish descent in the Copenhagen studies and mainly of European decent in the MAGIC and CHARGE, hereby reducing population stratification bias. However, due to the homogenous study population, our results may not necessarily apply to other ethnicities. There was a considerable overlap between the datasets (MAGIC and CHARGE) used for the two-sample MR analyses, which may bias the estimates in the direction of the observational estimates (and in the same direction any potential bias in the one-sample MR analyses) [53]. However, the F statistic indicated sufficient strength of both genetic instruments in one-sample analyses, and the risk of a biased null finding due to overlapping samples in the two-sample analyses is low. MR Egger analyses did not indicate pleiotropy and Cochran’s Q test suggested low heterogeneity. Plasma glucose is in the Copenhagen Studies measured in the non-fasting state. However, the association between genetic glucose variants and plasma glucose concentrations were similar for the Copenhagen Studies and in MAGIC using fasting glucose (Supplementary Tables 7 and 8), and a similar stepwise effect is seen using haemoglobin A1c [54].

In conclusion, in this observational and bidirectional one- and two-sample Mendelian randomization study, we found that elevated concentrations of plasma CRP and glucose are predictors of IHD and CVD death in the general population. We found no genetic association between CRP and glucose, or vice versa, suggesting that lowering glucose pharmacologically does not have a direct effect on low-grade inflammation.

### Electronic supplementary material

Below is the link to the electronic supplementary material.

Supplementary Material 1

## Data Availability

Aggregated data from the Copenhagen City Heart Study and the Copenhagen General Population Study may be available on reasonable request to the corresponding author. Summary level data from the MAGIC and CHARGE consortia are publicly available.
