# Supplementary material for: Contributions of elevated CRP, hyperglycaemia, and type 2 diabetes to cardiovascular risk in the general population: observational and Mendelian randomization studies
Source: Cardiovasc Diabetol. 2024 May 10;23:165. doi: 10.1186/s12933-024-02207-0 (PMC11088022; doi:10.1186/s12933-024-02207-0)
Supplement: Supplementary file 1 — Supplementary Material 1 [file 12933_2024_2207_MOESM1_ESM.docx]

**Supplemental Material**

**Contributions of CRP, glycemia, and type 2 diabetes to the risk of cardiovascular events in the general population: Observational and Mendelian randomization studies**

Monica G Rolver, MS^1,2^, Frida Emanuelsson, MD, PhD,^1,2^_,_ Børge G Nordestgaard, MD, DMSc^2,3,4^, Marianne Benn, MD, DMSc, PhD^1,2^

^1^Department of Clinical Biochemistry, Copenhagen University Hospital - Rigshospitalet, Blegdamsvej 9, 2100 Copenhagen, Denmark; ^2^Institute of Clinical Medicine, Faculty of Health and Medical Sciences, University of Copenhagen, Blegdamsvej 3B, 2200 Copenhagen, Denmark; ^3^Department of Clinical Biochemistry and ^4^The Copenhagen General Population Study, Copenhagen University Hospital - Herlev Gentofte, Borgmester Ib Juuls Vej 1, 2730 Herlev, Denmark

Contents

[**Supplementary Table 1** 2](#_Toc160191694)

[**Supplementary Table 2** 3](#_Toc160191695)

[**Supplementary Table 3** 4](#_Toc160191696)

[**Supplementary Table 4** 5](#_Toc160191697)

[**Supplementary Table 5** 6](#_Toc160191698)

[**Supplementary Table 6** 7](#_Toc160191699)

[**Supplementary Table 7** 9](#_Toc160191700)

[**Supplementary Table 8** 10](#_Toc160191701)

[**Supplementary Figure 1** 11](#_Toc160191702)

[**Supplementary Figure 2** 13](#_Toc160191703)

[**Supplementary Figure 3** 14](#_Toc160191704)

[**Supplementary Figure 4** 15](#_Toc160191705)

# **Supplementary Table 1**

| **Cohort name** | **Abbreviation** | **N** | **%, CHARGE** | **%, MAGIC** |  |
| --- | --- | --- | --- | --- | --- |
| Avon Longitudinal Study of Parents and their Children | ALSPAC | 4,099 | 2.01 | 2.48 |  |
| The Heredity and Phenotype Intervention Heart Study | HAPI | 868 | 0.42 | 0.52 |  |
| The Atherosclerosis Risk in Communities study | ARIC | 15,972 | 7.81 | 9.65 |  |
| Baltimore Longitudinal Study of Aging | BLSA | 1,231 | 0.60 | 0.74 |  |
| Busselton Health Study | BHS | 1,259 | 0.62 | 0.76 |  |
| Cardiovascular Health Study | CHS | 5,888 | 2.88 | 3.56 |  |
| Cohorte Lausannoise | CoLaus | 6,188 | 3.03 | 3.74 |  |
| Epidemiologic data on the syndrome of insulin resistance | DESIR | 708 | 0.35 | 0.43 |  |
| Fenland Study |  | 8,000 | 3.91 | 4.83 |  |
| Framingham Heart Study | FHS | 14,428 | 7.06 | 8.72 |  |
| InCHIANTI study |  | 1,202 | 0.59 | 0.73 |  |
| Cooperative Health Research in the Region of Augsburg, Southern Germany | KORA | 18,000 | 8.81 | 10.87 |  |
| METabolic Syndrome In Men | METSIM | 10,197 | 4.99 | 6.16 |  |
| Microisolates in South Tyrol | MICROS | 1,175 | 0.57 | 0.71 |  |
| Netherlands Twin Register | NTR | 9,530 | 4.66 | 5.76 |  |
| Orkney Complex Disease Study | ORCADES | 2,100 | 1.03 | 1.27 |  |
| Prospective Investigation of the Vasculature in Uppsala Seniors | PIVUS | 1,000 | 0.49 | 0.60 |  |
| Precocious Coronary Artery Disease Study | Procardis | 1,552 | 0.76 | 0.94 |  |
| The Rotterdam study |  | 10,994 | 5.38 | 6.64 |  |
| SardiNIA study |  | 6,921 | 3.39 | 4.18 |  |
| Twins UK |  | 11,000 | 5.38 | 6.65 |  |
| Uppsala Longitudinal Study of Adult Men | ULSAM | 7,273 | 3.56 | 4.39 |  |
| **Total** |  | **132,312** | **64.73%** | **79.94%** |  |

**The listed cohorts are credited as contributors to both** Meta-Analyses of Glucose and Insulin-related traits Consortium (MAGIC) and the Cohorts for Heart and Aging Research in Genomic Epidemiology (CHARGE) Inflammation Working Group (CIWG).

# **Supplementary Table 2**

Overview of the genetic instruments used for glucose and their association with glucose metabolism.

| Gene | Genomic coordinates (GRCh38) | Encoded gene product | Function/involvement in glucose metabolism | References for function | Tag SNP (Rsid) | Tag SNP position (GRCh38) | Type of variant |
| --- | --- | --- | --- | --- | --- | --- | --- |
| *G6PC2* | 2:168901291-168910000 | Islet-specific glucose-6-phosphatase catalytic subunit-related protein | Regulation of beta-cell glucose-stimulated insulin secretion | DOI:10.2337/db12-1067  DOI: 10.1007/s40618-020-01483-3  DOI: 10.1016/j.jbc.2021.101534 | rs560887 | 2:168906638 | Intron variant |
| *GCK* | 7:44143213-44189439 | Glucokinase | Beta-cell glucose metabolism, regulation of glucose-stimulated insulin secretion | DOI: 10.1186/s13059-023-02935-8  DOI: 10.1016/j.tem.2022.12.007  DOI: 10.2337/diab.39.6.647 | rs4607517 | 7:44196069 | Intron variant |
| *DGKB* | 7:14145049-14974858,  7: 15200317-15,562015 | Diacylglycerol kinase beta | Cell signalling, regulation of glucose-stimulated insulin secretion | DOI: 10.1007/s00125-010-1753-5  DOI: 10.1038/s41467-020-18581-8  DOI: 10.1210/en.2013-1356 | rs2191349 | 7:15024684 | Intergenic variant |
| *ADCY5* | 3:123282296-123449090 | Adenylate cyclase 5 | Glucose signalling and regulation of insulin secretion, proinsulin-to-insulin conversion | DOI:10.2337/db13-1607  DOI:10.1371/journal.pone.0023639  DOI:10.2337/db17-0464 | rs11708067 | 3:123346931 | Intron variant |
| *CDKN2A/B* | 9:21967752-21995324 (A),  9:22002903-22009313 (B) | Cyclin-dependent kinase inhibitor 2A (p16)  Cyclin-dependent kinase inhibitor 2B (p15) | Beta-cell mass and function, first phase glucose-induced insulin secretion | DOI:10.1016/j.tem.2015.01.008  DOI:10.1007/s00125-010-2038-8  DOI:10.2337/db09-0736  DOI:10.1152/ajpendo.00496.2010 | rs10811661 | 9:22134095 | Upstream intergenic variant |
|  |  |  |  |  | rs2383206 | 9:22115027 | Intron variant |
| *TCF7L2* | 10:112950247-113167678 | Transcription factor 7-like 2 | Transcription factor, regulatory function of GLP-1 induced insulin response, beta-cell function | DOI: 10.1172/JCI30706  DOI: 10.2337/db20-0573  DOI: 10.1007/s00125-007-0753-6 | rs7903146 | 10:112998590 | Intron variant |

Modified from Emanuelsson et al, DOI: 10.1007/s00125-023-06054-8

### **Supplementary Table 3**

| **Genetic instruments, glucose** | | | **Copenhagen studies** | | | | | | **CHARGE** | | | **MAGIC** | | |
| --- | --- | --- | --- | --- | --- | --- | --- | --- | --- | --- | --- | --- | --- | --- |
| **Gene** | **Variant** | | **N** | **β_CRP_ (SE)** | **P β_CRP_** | **N** | **β_glucose_ (SE)** | **P β_glucose_** | **N** | **β_CRP_ (SE)** | **P β_CRP_** | **N** | **β_glucose_ (SE)** | **P β_glucose_** |
| *G6PC2/ABCB11* | | rs560887 | 110,052 | 0.02 (0.023) | 0.472 | 110,058 | 0.08 (0.006) | 1.95×10^-131^ | 204,402 | -0.003 (0.004) | 0.49267 | 198,018 | 0.075 (0.002) | 0 |
| *DGKB* | | rs2191349 | 110,112 | -0.01 (0.021) | 0.590 | 110,128 | 0.03 (0.005) | 3.96×10^-12^ | 204,402 | -0.0002 (0.004) | 0.94911 | 197,832 | 0.258 (0.002) | 0 |
| *GCK* | | rs4607517 | 110,104 | 0.06 (0.030) | 0.027 | 110,110 | 0.06 (0.007) | 9.24×10^-40^ | 204,402 | 0.004 (0.005) | 0.45445 | 200,392 | 0.061 (0.002) | 0 |
| *TCF7L2* | | rs7903146 | 107,758 | -0.01 (0.024) | 0.560 | 107,764 | 0.05 (0.006) | 1.50 ×10^-14^ | 204,402 | 0.001 (0.004) | 0.87058 | 199,170 | 0.026 (0.002) | 2.00×10^-35^ |
| *CDKN2A/B* | | rs10811661 | 108,183 | -0.01 (0.029) | 0.668 | 108,189 | 0.03 (0.007) | 2.36 ×10^-6^ | 204,402 | -0.006 (0.005) | 0.24876 | 196,734 | 0.022 (0.002) | 1.59×10^-24^ |
| *ADCY5* | | rs11708067 | 110,217 | -0.03 (0.025) | 0.212 | 110,223 | 0.04 (0.006) | 7.13×10^-16^ | 204,402 | 0.012 (0.004) | 0.01491 | 199,932 | 0.028 (0.002) | 0 |
| Weighted allele score | |  | 106,452 | 0.15 (0.200) | 0.455 | 106,458 | 0.99 (0.047) | 1.19 ×10^-189^ |  |  |  |  |  |  |
| **Genetic instruments,**  **C-reactive protein** | | | **Copenhagen studies** | | | | | | **CHARGE** | | | **MAGIC** | | |
| **Gene** | **Variant** | | **N** | **β_CRP_ (SE)** | **P β_CRP_** | **N** | **β_glucose_ (SE)** | **P β_glucose_** | **N** | **Β_CRP_ (SE)** | **P β_CRP_** | **N** | **β_glucose_ (SE)** | **P β_glucose_** |
| *CRP* | | rs1205 | 105,828 | 0.31 (0.023) | 1.36×10^-192^ | 105,834 | -0.01 (0.005) | 0.271 | 204,402 | 0.18 (0.004) | 1.30×10^-305^ | 197,610 | 0.0003 (0.0017) | 0.9326 |
| *CRP* | | rs1130864 | 105,845 | 0.29 (0.023) | 3.27×10^-122^ | 105,821 | -0.003 (0.056) | 0.553 | 204,402 | 0.14 (0.004) | 1.95×10^-201^ | 166,896 | -0.0005 (0.0019) | 0.6037 |
| *CRP* | | rs3093077 | 108,002 | 0.45 (0.049) | 1.13×10^-75^ | 108,008 | -0.006 (0.012) | 0.581 | 204,402 | 0.22 (0.007) | 1.30×10^-265^ | 165,518 | -0.0006 (0.0038) | 0.6893 |
| Weighted allele score | |  | 105,759 | 2.85 (0.159) | 5.65×10^-281^ | 105,765 | -0.04 (0.038) | 0.275 |  |  |  |  |  |  |

Genetic variants used as instruments for glucose (upper panel) and C-reactive protein (CRP; lower panel) and their association with CRP and non-fasting plasma glucose in the Copenhagen studies, with CRP in CHARGE(1) and with fasting plasma glucose in MAGIC(2). CRP values was transformed to natural log in both cohorts (Copenhagen studies and CHARGE).

N = number, β = beta-coefficient, SE = standard error of the beta-coefficient, P = p-value of the beta-coefficient.

### **Supplementary Table 4**

| **Genetic instruments, glucose** | | **Copenhagen studies** | | **CHARGE** | **MAGIC** |
| --- | --- | --- | --- | --- | --- |
| Gene | Variant | Individuals with available genotype | Individuals with missing genotype | Individuals with available genotype | Individuals with available genotype |
| *G6PC2/ABCB11* | rs560887 | 110,058 | 2,757 | 204,402 | 198,018 |
| *DGKB* | rs2191349 | 110,128 | 2,687 | 204,402 | 197,832 |
| *GCK* | rs4607517 | 110,110 | 2,705 | 204,402 | 200,392 |
| *TCF7L2* | rs7903146 | 107,764 | 5,051 | 204,402 | 199,170 |
| *CDKN2A/B* | rs10811661 | 108,189 | 4,626 | 204,402 | 196,734 |
| *ADCY5* | rs11708067 | 110,223 | 2,592 | 204,402 | 199,932 |
| *Weighted allele score* |  | 106,458 |  |  |  |
| **Genetic instruments, C-reactive protein** | | **Copenhagen studies** | | **CHARGE** | **MAGIC** |
| Gene | Variant | Individuals with available genotype | Individuals with missing genotype | Individuals with available genotype | Individuals with available genotype |
| *CRP* | rs1205 | 105,834 | 6,981 | 204,402 | 197,610 |
| *CRP* | rs1130864 | 105,851 | 6,964 | 204,402 | 166,896 |
| *CRP* | rs3093077 | 108,008 | 4,807 | 204,402 | 165,518 |
| *Weighted allele score* |  | 105,759 |  |  |  |

Specification of number of individuals with available genotypes in the Copenhagen studies, CHARGE(1), and MAGIC(2). Number of individuals used to create the weighted allele score and individuals with missing genotypes and is also specified for the Copenhagen studies.

### **Supplementary Table 5**

| **Diagnosis** | **ICD-8 codes** | **ICD-10 codes** |
| --- | --- | --- |
| Type 2-diabetes | 250 | E11, E13, E14 |
| Ischemic heart disease | 410 – 414 | I20 – I25 |
| Cardiovascular death | 410 – 414, 427.09 – 427.11,  433 – 434, 440, 443.99, 445 | I20 – I25, I50.0 – I50.9, I63,  I70, I739, E105, E115, E145 |

International Classification of Diseases (ICD) codes used to define type 2 diabetes, ischemic heart disease, and cardiovascular death. ICD codes were obtained from the national Danish Patient Registry and national Danish Causes of Death Registry, with data on all patient in-hospital visits from January 1^st^, 1977, through December 13^th^, 2018. From 1995, data from emergency wards and outpatient clinics was also included. ICD-9 was not used as this revision of ICD was never introduced in Denmark, and ICD-10 replaced ICD-8 as of January 1^st^, 1994.

### **Supplementary Table 6**

| **Variable** | **All** | **No diabetes** | **Type 2 diabetes** |  |
| --- | --- | --- | --- | --- |
| Number of individuals | 112,810 | 106,554 (94%) | 6,256 (6%) |  |
| Age, years | 58 (48-67) | 57 (47-67) | 65 (58-72) |  |
| Sex |  |  |  |  |
| Men | 61,799 (55%) | 59,111 (55%) | 2,688 (43%) |  |
| Women | 51,011 (45%) | 47,443 (45%) | 3,568 (57%) |  |
| Body mass index, kg/m^2^ | 25.6 (23.2-28.4) | 25.4 (23.1-28.2) | 29.2 (26.3-32.8) |  |
| Alcohol, units/week | 8 (3-15) | 8 (3-15) | 7 (2-15) |  |
| Tobacco | 15 (5-30) | 15 (5-30) | 26 (11-43) |  |
| Never smoked | 42,891 (43%) | 41,298 (43%) | 1,693 (33%) |  |
| Former smoker | 41,414 (40%) | 39,064 (40%) | 2,350 (45%) |  |
| Current smoker | 17,475 (17%) | 16,354 (17%) | 1,121 (22%) |  |
| Physical activity |  |  |  |  |
| Low | 33,525 (30%) | 30,845 (29%) | 2,680 (43%) |  |
| Moderate | 76,301 (68%) | 72,906 (69%) | 3,395 (55%) |  |
| High | 2,651 (2%) | 2,532 (2%) | 119 (2%) |  |
| Systolic blood pressure, mmHg | 135 (122-150) | 135 (121-150) | 142 (130-158) |  |
| Diastolic blood pressure, mmHg | 80 (72-88) | 80 (72-88) | 81 (74-90) |  |
| Hypertension | 62,326 (55%) | 57,154 (54%) | 5,172 (83%) |  |
| Glucose, mmol/L | 5.2 (4.7-5.7) | 5.1 (4.7-5.7) | 6.3 (5.4-7.7) |  |
| C-reactive protein, mg/L | 2.5 (1.0-2.3) | 2.4 (0.9-2.2) | 4.1 (1.3-4.2) |  |
| HDL cholesterol, mmol/L | 1.5 (1.2-1.9) | 1.5 (1.2-1.9) | 1.2 (1.0-1.6) |  |
| LDL cholesterol, mmol/L | 3.2 (2.6-3.9) | 3.2 (2.6-3.9) | 2.8 (2.1-3.7) |  |
| Triglycerides, mmol/L | 1.4 (1.0-2.1) | 1.4 (1.0-2) | 1.96 (1.3-2.9) |  |

Baseline characteristics for individuals in the Copenhagen City Hearts Study and Copenhagen General Population Study shown for all individuals and by diabetes status. Data are absolute numbers (%) for categorical variables and median (interquartile range) for continuous variables. Glucose measurements were performed on non-fasting samples. Units of alcohol per week is only fo r individuals currently drinking alcohol. Physical activity, tobacco consumption, and use of lipid-lowering medication was self-reported. Diagnoses of type 2 diabetes and hypertension were defined by International Classification of Disease (ICD)-10 and 8 codes was obtained from the national Danish Patient Registry.

| **Analysis** | **Beta-coefficient** | **95% Confidence Interval** | | **P-value** | | **P value MR Egger intercept** | | **I^2^** | | **P value Cochran’s Q test** | |
| --- | --- | --- | --- | --- | --- | --- | --- | --- | --- | --- | --- |
| *Outcome: Glucose* | | |  | |  | |  | |  | |  |
| MR Egger IVW | -0.0005 | -0.142, 0.013 | | 0.946 | |  | | 0.0% | | 0.94 | |
| MR Egger | 0.007 | -0.082, 0.095 | | 0.883 | | 0.873 | |  | |  | |
| MR Median | -0.003 | -0.019, 0.014 | | 0.750 | |  | |  | |  | |
| *Outcome: CRP* | | |  | |  | |  | |  | |  |
| MR Egger IVW | -0.034 | -0.126, 0.058 | | 0.467 | |  | | 33.5% | | 0.18 | |
| MR Egger | 0.051 | -0.151, 0.252 | | 0.623 | | 0.354 | |  | |  | |
| MR Median | -0.022 | -0.136, 0.091 | | 0.699 | |  | |  | |  | |

### **Supplementary Table 7**

Association of genetically determined plasma C-reactive protein (CRP) and glucose concentrations in two-sample Mendelian Randomization, using publicly available summary data from the MAGIC and CHARGE consortia. Inverse-variance weighted (IVW), MR-Egger and weighted median analyses were performed to study causality between genetically determined plasma CRP concentrations to plasma glucose and *vice versa*. Pleiotropy/heterogeneity was tested for using Cochran’s Q test and reported as I^2^ where an I^2^ between 0-40% is considered low, between 40-60% as moderate and above 60% as high(3).

| **Analysis** | **Beta-coefficient** | **95% Confidence Interval** | **P-value** | **P value MR Egger intercept** | **I^2^** | **P value Cochran’s Q test** |
| --- | --- | --- | --- | --- | --- | --- |
| *Outcome: Glucose* |  |  |  |  |  |  |
| MR Egger IVW | -0.027 | -0.054, -0.000 | 0.049 |  | 0% | 0.83 |
| MR Egger | 0.028 | -0.154, 0.209 | 0.765 | 0.55 |  |  |
| MR Median | -0.013 | -0.057, 0.030 | 0.546 |  |  |  |
| *Outcome: CRP* |  |  |  |  |  |  |
| MR Egger IVW | 0.123 | -0.309, 0.554 | 0.578 |  | 20.9% | 0.28 |
| MR Egger | 0.896 | -0.184, 1.978 | 0.104 | 0.13 |  |  |
| MR Median | -0.267 | -0.847, 0.314 | 0.368 |  |  |  |

### **Supplementary Table 8**

As a sensitivity analysis, association of genetically determined plasma C-reactive protein (CRP) and glucose concentrations were examined in two-sample Mendelian randomization, using data from the Copenhagen studies only, well knowing that there is a 100% overlap between the individuals used for estimating the association with glucose and the association with CRP. Inverse-variance weighted (IVW), MR-Egger, and weighted median analyses were performed to study causality between genetically determined plasma CRP concentrations to plasma glucose and *vice versa*. Pleiotropy/heterogeneity was tested for using Cochran’s Q test and reported as I^2^ where an I^2^ between 0-40% is considered low, between 40-60% as moderate and above 60% as high(3).

# **
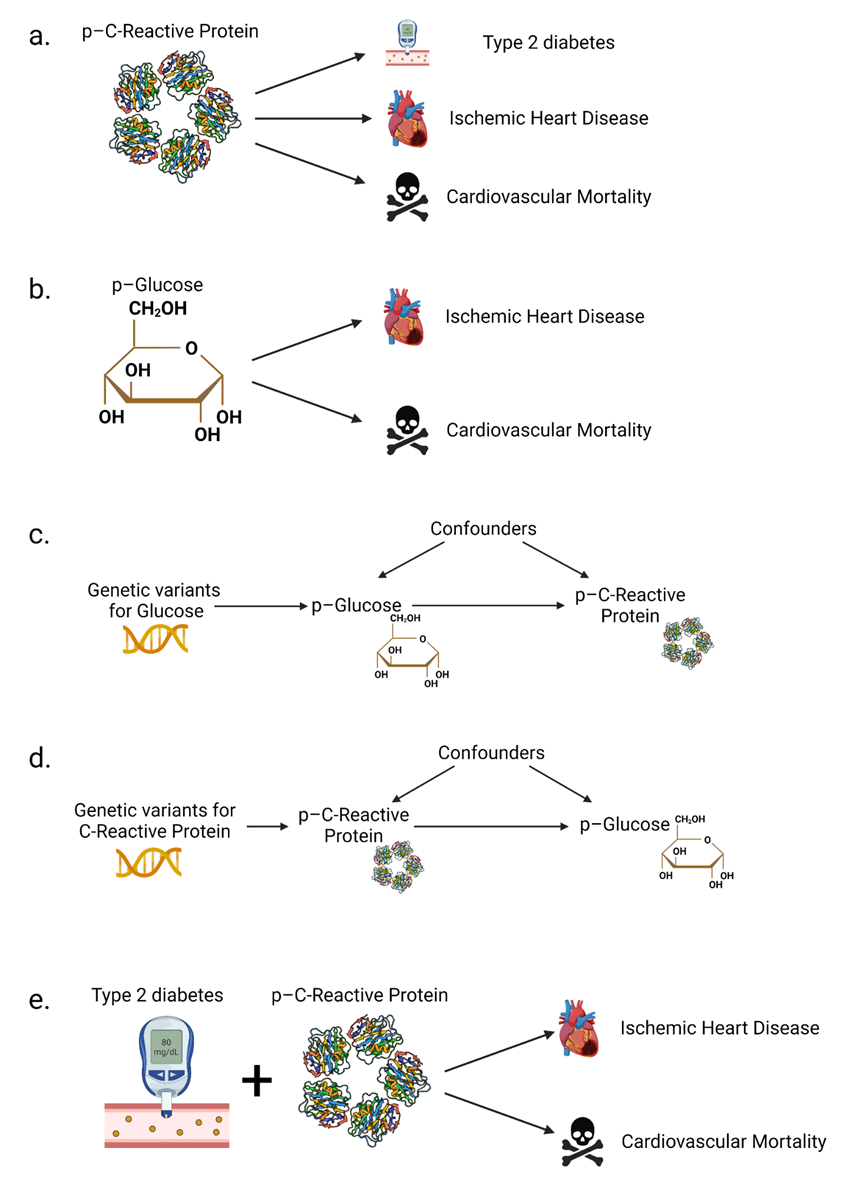
Supplementary Figure 1**

Graphic illustrations of the aims and hypotheses of the study.
**a.** Associations of plasma C-reactive protein (CRP) concentrations and incidence of type 2 diabetes (T2D) and risk of ischemic heart disease (IHD) and cardiovascular disease death (CVD death).
**b.** Associations of plasma glucose concentrations and risk of IHD and CVD death.
**c and d.** Direct acyclic graphs illustrating the hypothesized causal pathways between plasma concentrations of CRP and glucose. To perform a Mendelian randomization analysis, there are three main assumptions: 1) the genotype should be associated with the exposure (glucose / CRP), 2) the genotype should not be associated with confounders of the exposure – outcome association, and 3) the genotype should only be associated with the outcome through the exposure. Horizontal lines signify the relationships tested in bidirectional Mendelian Randomization. Genetic variants were used to assess the causal relationship from glucose to CRP (upper part) and vice versa (lower part).
**e.** Cumulative incidence of IHD and CVD death stratified by T2D and plasma CRP concentrations.

# **Supplementary Figure 2**


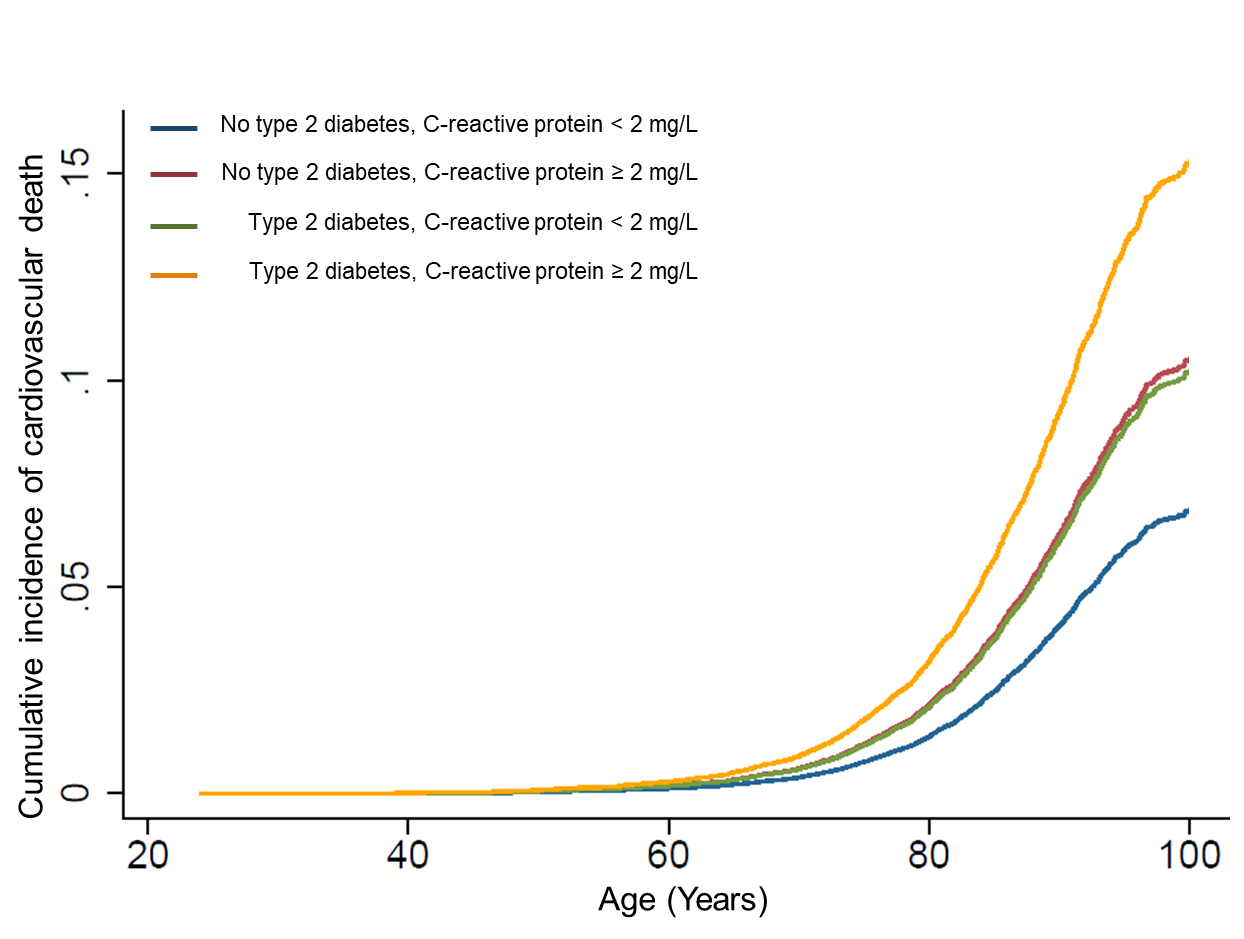


Cumulative incidence of cardiovascular death calculated using competing risk regression based on Fine and Gray proportional sub-hazards model to account for the possibility of death from other cause as competing event. T2D = Type 2 diabetes, CRP = C-reactive protein.

# **Supplementary Figure 3**

**
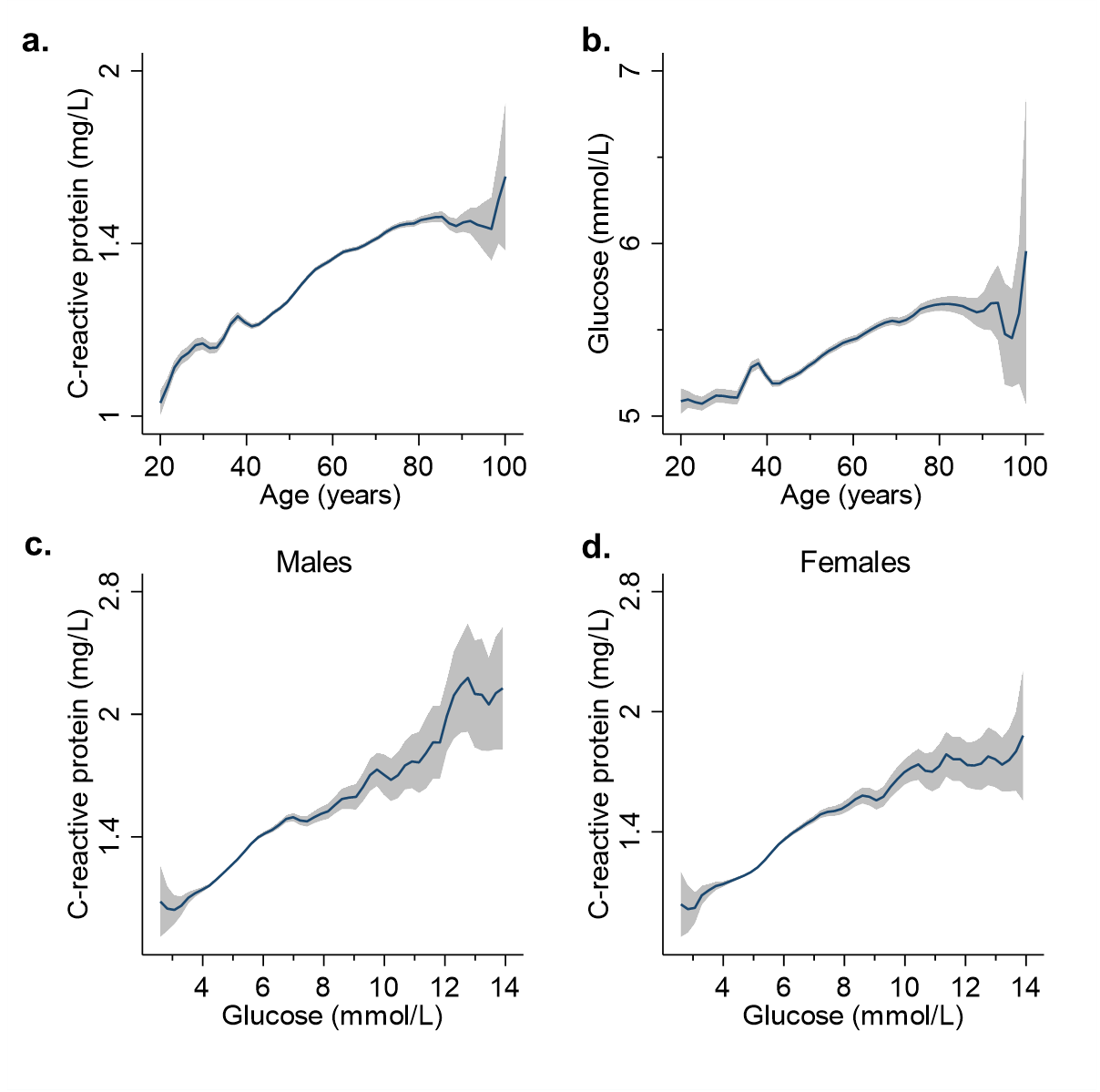
**

Associations between plasma CRP, glucose concentrations, age, and sex.

**a.** Plasma CRP as a function of age (blue line) with 95% CI (grey area). Plasma CRP concentration shown as logarithmic scale on y-axis.

**b.** Plasma glucose as a function of age (blue line) with 95% CI (grey area).

**c.** Association between plasma CRP and glucose in males.

**d.** Association between plasma CRP and glucose in females. The observational associations between plasma CRP and glucose concentrations were investigated using multifactorially adjusted linear regressions and graphically displayed using kernel-weighted local polynomial smoothing and geometric means with 95% CIs. 1 mmol/L glucose is equivalent to 18 mg/dL.

# **Supplementary Figure 4**


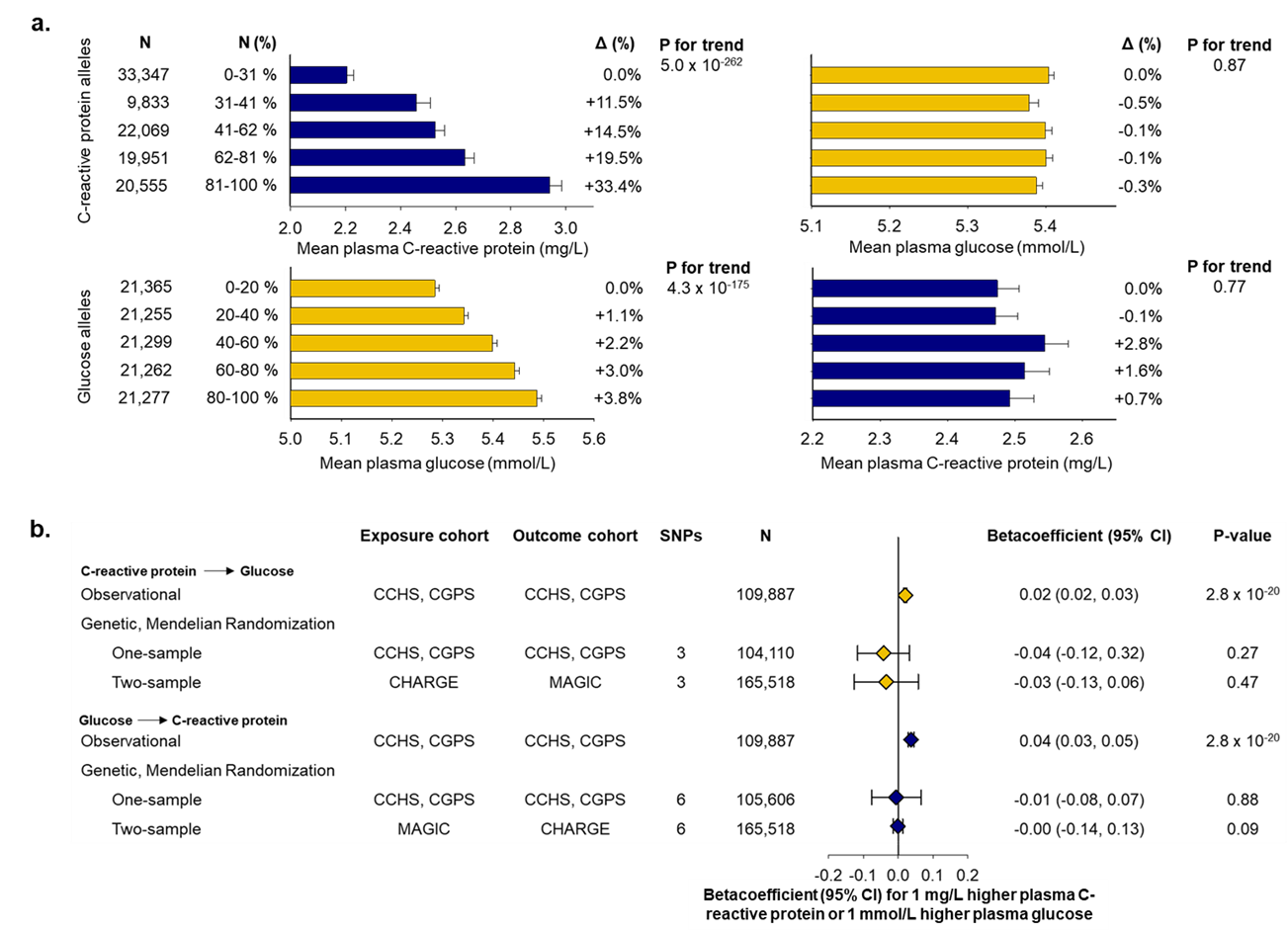


Mean plasma C-reactive protein (CRP) and glucose concentrations as a function of, respectively, the weighted CRP and the glucose allele scores as quintiles. The weighted CRP allele score was generated using the genetic variants rs1205, rs1130864, and rs3093077, located in the *CRP* gene. The weighted glucose allele score was generated using the genetic variants rs560887, rs2191349, rs4607517, rs7903146, rs10811661, and rs11708067.

**References**

1. Ligthart S, Vaez A, Võsa U, Stathopoulou MG, de Vries PS, Prins BP, et al. Genome Analyses of &gt;200,000 Individuals Identify 58 Loci for Chronic Inflammation and Highlight Pathways that Link Inflammation and Complex Disorders. Am J Hum Genet [Internet]. 2018 Nov;103(5):691–706. Available from: https://linkinghub.elsevier.com/retrieve/pii/S0002929718303203

2. Chen J, Spracklen CN, Marenne G, Varshney A, Corbin LJ, Luan J, et al. The trans-ancestral genomic architecture of glycemic traits. Nat Genet. 2021 Jun;53(6):840–60.

3. Higgins JPT. Measuring inconsistency in meta-analyses. BMJ [Internet]. 2003 Sep 6;327(7414):557–60. Available from: http://link.springer.com/10.1007/s10844-006-2974-4
